# Non-invasive preimplantation genetic testing: a literature review

**DOI:** 10.5935/1518-0557.20210102

**Published:** 2022

**Authors:** Larissa Nogueira Sousa, Paula Bruno Monteiro

**Affiliations:** 1 Centro Universitário Christus, Fortaleza, Ceará, Brazil

**Keywords:** biopsy, genetic testing, aneuploidy, assisted reproduction

## Abstract

Non-invasive preimplantation genetic testing emerged from the discovery of embryonic DNA in spent embryo culture medium. Considering that such methodology would be an important advance in assisted reproduction, this study aimed to evaluate the current scientific evidence, based on the reliability of non-invasive chromosome screening, through a literature review. We analyzed 14 original research papers in PubMed and SciELO, in English and Portuguese, published between 2016 and 2021 related to the topic. The agreement rate for ploidy compared to the traditional method ranged from 3.5% - 93.8% raising the discussion about the possible causes of this large variation, which may be due to the day of collection, spent culture media contamination, amplification methodology or the cytogenetic method used by each author. We concluded that the non-invasive test has many advantages over the traditional method, but that clinical replacement is not yet possible, and further studies are needed in order to have an accurate clinical test with the non-invasive methodology.

## INTRODUCTION

Preimplantation genetic testing (PGT) is defined as the multidisciplinary clinical application of genetic technologies and assisted reproduction techniques aimed at examining cells from an embryo in its *in vitro* developmental phase (Zegers-Hochschild *et al*., 2017). Such techniques began to be used for genetic screening, with the purpose of trying to improve pregnancy rates in certain groups of patients with poor IVF-procedure prognosis, such as those with advanced maternal age, recurrent miscarriages and repeated deployment failures. The test is done through an embryo biopsy, and one can remove the polar bodies, blastomere or trophectoderm (Almeida *et al*., 2013; Soeiro, 2012).

The trophectoderm biopsy is the most used, as it can be performed from the fifth day of embryonic development - blastocyst stage, in which the embryo is more developed, thus having more material for analysis, yielding more precision to the result (Homer, 2019). However, it is an invasive procedure, and it may compromise embryo viability and implantation potential, and it adds potential concerns about long-term effects on the offspring, which are very difficult to assess. In addition, the procedure for performing the biopsy requires considerable training and experience to manipulate the embryo, increasing the costs of performing the technique (Xu *et al*., 2016).

Thus, with the discovery of free embryonic DNA (cfeDNA) found in both blastocele fluid (BF) and spent culture medium (SCM) of the embryo, the possibility of performing a non-invasive preimplantation genetic test (NiPGT), eliminating the need for embryo biopsy, avoiding the risks related to the invasive procedure, with a simpler and potentially more affordable methodology, in addition to enabling the test in embryos with non-viable morphology for biopsy, with a better representation of the entire embryo. Currently, there are two main research approaches for collecting cfeDNA for aneuploidy testing, they are: collection of spent culture medium, and combining BF collection with spent culture medium (Kuznyetsov *et al*., 2018; Stigliani *et al*., 2014).

Considering that the NiPGT methodology would be an important advance in assisted reproduction, this study aimed to evaluate the current scientific evidence based on the reliability of non-invasive chromosome screening, using extra embryonic DNA and its clinical potential.

## MATERIALS AND METHODS

**Figure 1:**
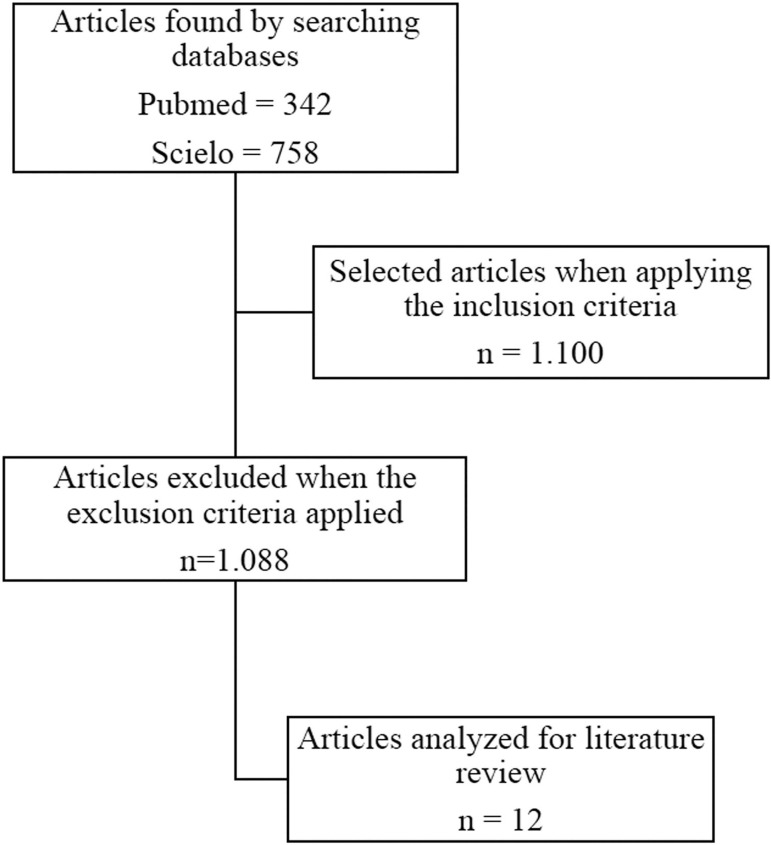
Flowchart of the methodology used in this study.

This is a literature review, aimed to address the techniques of invasive and non-invasive preimplantation genetic testing methods, evaluating the advantages and disadvantages of both techniques, bringing together current studies on advances in non-invasive testing, highlighting the level of reliability and its clinical potential. Data collection began in August 2020 and was carried out until May 2021, in the scientific databases PubMed and SciELO (Figure 1), using the keywords: biopsy, genetic testing, aneuploidy and assisted reproduction. We found 1,100 papers and analyzed 12 original articles that assess the NiPGT methodology, published between 2015 and 2021, in English and Portuguese. Tests using animals, monographs, case reports and review articles were not part of the results. The analyzed data were organized in a table using the Microsoft Excel^©^ platform.

## RESULTS

Among the 12 studies analyzed (Table 1), only Feichtinger *et al*. (2017) did not use trophectoderm biopsy to validate their experiment. The analysis was performed by comparing spent culture media with polar body biopsy, which brought limitations to the analysis, such as lack of paternal chromosomal information. While the other authors used analytes in addition to trophectoderm biopsy (Kuznyetsov *et al*., 2018; Ho *et al*., 2018; Huang *et al*., 2019; Yin *et al*., 2021; Rubio *et al*., 2019; Yeung *et al*., 2019; Jiao *et al*., 2019), such as blastocele fluid (Kuznyetsov *et al*., 2018; Jiao *et al*., 2019) and whole embryo (Xu *et al*., 2016; Kuznyetsov *et al*., 2018; Ho *et al*., 2018; Huang *et al*., 2019). The agreement rate between spent culture medium (SCM) and polar body biopsy was 27%, lower than the SCM analysis with blastocele fluid, which achieved an agreement of 87.5% (Kuznyetsov *et al*., 2018). For those who chose to correlate the spent culture medium with the trophectoderm biopsy, the agreement rate ranged from 3.5% - 93.8% raising the discussion about the possible causes for this large variation, which may be due to the day of collection of spent culture media, media contamination, amplification methodology or cytogenetic method used by each author.

**Table 1.** Studies that used spent culture medium to validate NiPGT.

	Samples			Analyze		
	Development Day	Number of samples	culture medium	Agreement	Amplification Kits	Cytogenetics Method
**Spent culture medium** Shamonki *et al*. (2016) Xu * et al*. (2016) Feichtinger *et al*. (2017) Liu *et al.* (2017) Ho *et al*. (2018) Huang *et al*. (2019) Vera-Rodriguez *et al*. (2018) Yin *et al*. (2021) Rubio *et al*. (2019) Yeung *et al.* (2019)	D3-5/6 D3-5 D1-5 D1-5 D3-5 D5/6 D3-5 D5 D4-5/6/7 D3-5/6	57 42 22 88 41 52 56 75 115 168	two steps two steps single step single step single step two steps two steps two steps two steps two steps	3.5% 85.7% 27% 64.5% D3-56.3%/D5-65% 93.8% 30.4% 27.2% D5-78.7%/D6/7- 84% 62.1%	Repli-G MALBAC SurePlex MALBAC Picoplex MALBAC SurePlex ChromInst ThermoFisher SurePlex	CGH NGS CGH NGS NGS NGS CGH NGS NGS NGS
**Spent culture medium and blastocoel fluid** Kuznyetsov *et al*. (2018) Jiao *et al*. (2019)	D5-6/7 D5/6	47 62	two steps two steps	87.5% 76.1%	SurePlex MALBAC	NGS NGS

Main studies reporting the use of spent culture medium for preimplantation genetic testing. D1-7, day of embryonic development; NGS, next-generation sequencing; CGH, comparative genomic hybridization.

The choice to collect day 3 and day 5/6 developmental culture medium was because this is a convenient time point in the laboratory evaluation of embryos. Analysis of the two-stage culture medium was performed by 75% of the studies, while the other authors used a single-stage culture medium (Feichtinger *et al*., 2017; Liu *et al*., 2017; Ho *et al*., 2018). Regarding the methods of analysis of the collected material, SCM, trophectoderm biopsy and of polar bodies went through the same protocol. Of the selected studies, 33% used Sureplex as a method of DNA amplification. For chromosomal analysis, the most used cytogenetic method was Next Generation Sequencing (NGS).

## DISCUSSION

Hammond *et al*. (2016) demonstrated higher levels of DNA in culture media that were exposed to embryos compared to culture media controls, suggesting that much of the genetic material detected in spent culture media is of embryonic origin. From this discovery, emerged studies and variations regarding the collection of SCM.

The length of time an embryo maintains contact with the enriched medium depends on the standard operating procedures of the IVF laboratory. While some laboratories cultivate embryos in a single-stage, monophasic medium (embryo is cultured in the same drop in the middle from day 1 to day 5/6), others chose to sequentially change the culture medium once or twice between fertilization and blastulation (Wu *et al*., 2015).

Studies using single-stage culture medium hypothesized that this protocol could increase the level of excreted DNA, due to greater exposure of the embryo in the SCM. Feichtinger *et al*. (2017) and Liu *et al*. (2017) demonstrated amplification rates of samples from the culture medium of 81.8% and 90.9%, with an average DNA yield of 21.33 ng/µl and 25 ng/µl, respectively. However, cfDNA degradation may increase over time, decreasing cfDNA quality (Feichtinger *et al*., 2017; Liu *et al*., 2017; Ho *et al*., 2018).

Shamonki *et al*. (2016) and other authors (Xu *et al*., 2016; Kuznyetsov *et al*., 2018; Huang *et al*., 2019; Vera-Rodriguez *et al*., 2018; Yin *et al*., 2021; Rubio *et al.,* 2019; Yeung *et al*., 2019; Jiao *et al*., 2019) used the methodology of embryo cultivation in a two-stage media, pointing out a hypothesis that the exchange of culture media would decrease the rate of maternal contamination and the degradation of embryonic DNA. Rubio *et al*. (2019) found in their study that the longer the embryo remains in specific culture conditions, the greater the specificity of the test, with no significant impact on sensitivity. This statement came from their results that showed that the false positive (FP) and false negative (FN) values were better when considering day 6/7 of the SCM, with only 8.6% FP and 2.5% FN and increase sensitivity and specificity of 95.2% and 82.1%, respectively; compared to the analysis made on day 4/5 of the SMC, where 13.9% were FP and 2.8% FN, and the sensitivity and specificity were 94.5% and 71.7%, respectively.

It is extremely important to consider how the embryo is treated during its development, because it can determine not only the quantity and quality of cfDNA, but also, and more importantly, its origin. In addition to nuclear DNA, mitochondrial DNA (mtDNA) can also be detected in the embryo's culture medium (Stigliani *et al*., 2013; Wu *et al*., 2015). However, the secretion mechanism of this DNA is not fully understood. The hypothesis is that this genetic material can be released from cells undergoing apoptosis as part of a controlled elimination process. It is believed that the DNA of spent culture medium comes from both the inner cell mass (ICM) and the trophectoderm (TE) because both strains undergo apoptosis during pre-implantation development (Kuznyetsov *et al*., 2018; Ho *et al*., 2018; Wu *et al*., 2015).

The hypothesis that the DNA comes from the entire embryo brings advantages to NIPGT over the pre-implantation genetic test currently used, as although there seems to be a high rate of agreement between TE cells and ICM cells, a TE biopsy may not always represent the whole embryo(Kuznyetsov *et al*., 2018; McCoy, 2017). Studies that used the whole embryo as the analyte showed agreement rates with NiPGT ranging from 56.3% - 96.6%, the authors point out maternal contamination and embryo mosaicism as possible causes for this variability (Xu *et al*., 2016; Kuznyetsov *et al*., 2018; Ho *et al*., 2018; Huang *et al*., 2019).

Embryonic mosaicism is one of the main limitations of the PGT. It is defined as mitotic errors that appear in embryonic cells after the cleavage stage and form a mixture of euploid and aneuploid cells that can present in different ways, and when detected there is debate about embryo transfer or disposal (McCoy, 2017). Transfers can result in successful implantation and healthy births (Eggenhuizen *et al*., 2021; Greco *et al.,* 2015), but it can also result in decreased implantation, as well as an increased risk of genetic abnormalities and adverse pregnancy outcomes (Eggenhuizen *et al*., 2021).

Another point to consider is the risk that the genetic material detected in the spent culture medium is due to contamination, which can occur for several reasons, but the most discussed is the maternal contamination that can arise from cumulus cells that remain adhered to the zona pellucida after denudation, or the polar bodies after their extrusion from the oocyte (Hammond *et al*., 2016). An analysis using single nucleotide polymorphism (SNP) was performed to determine the ratio between embryonic and maternal DNA. The group used the alleles identified in the trophectoderm biopsies as a reference for the embryonic DNA haplotype; and, as a reference for maternal contamination they used alleles identified in the follicular fluid. The rate of partial and total maternal contamination was 60.8% (Vera-Rodriguez *et al*., 2018). To prevent this possible contamination, the authors suggest that cumulus cells be carefully washed (Xu *et al*., 2016; Huang *et al*., 2019). Yeung *et al*. (2019) included in their experiment an additional wash on day 3, before each embryo was transferred to its individual culture. While Jiao *et al*. (2019) demonstrated minimal or no maternal contamination, through the hypothesis that the use of cryopreserved blastocysts, due to thawing and variation in the concentration of reagents, helped eliminate cumulus cells.

Different whole genome amplification (WGA) protocols seem to affect cfDNA amplification success rates in SCM samples. The two methodologies most present in the studies are the Surelex/PicoPlex and Multi-Loop Based Amplification Cycles (MALBAC), which promise less amplification bias compared to SurePlex. According to a study in which the two techniques were compared, SurePlex proved to be more suitable for detecting changes in copy number than MALBAC, in which the amplified samples showed non-uniformity across the genome, leading to false positives (Deleye *et al*., 2015).

Studies have shown that modifications were needed in both types of methodology, such as the addition of six non-standard Sureplex amplification cycles, to ensure that the amount of DNA was sufficient (Ho *et al*., 2018). Improvements in MALBAC, which reduced the number of steps for library preparation of new primer designs from ten hours to two and a half hours, yielding high quality read rates (Jiao *et al*., 2019).

Regarding pre-implantation genetic screening methods, next-generation sequencing (NGS) and comparative genomic hybridization (CGH) are the most used, as they enable a complete genome analysis. NGS excels due to robust, high-throughput, customizable parallel analysis of multiple samples in a single sequencing run. A study comparing the two techniques using trophectoderm biopsy cells as samples provided clinical evidence that NGS detected all types of human blastocyst aneuploidies, segmental changes suggesting detection of partial aneuploidies or unbalanced translocations and mosaicism more accurately compared to mosaicism in the CGH screening (Yang *et al*., 2015).

A recent study compared the two chromosomal analysis techniques Veriseq (Illumina^®^) and NICS (Yikon^®^) when used in SCM, raising the question whether the disagreements in the NIPGT results are due to the techniques used. The results led to a similar diagnostic agreement (74.6% SCM-NICS *vs*. 72.3% SCM-Veriseq), suggesting that the disagreements are not due to technical limitations. As for the SCM sensitivity analyses, both techniques (78.0 for NICS and 80.0 for Veriseq) were higher than the specificity (69.7 for NICS and 60.6 for Veriseq); therefore, the authors suggest that the method is more effective in identifying embryos with chromosomal abnormalities than in selecting normal and transferable embryos (Lledo *et al*., 2021). Sensitivity values were similar to the studies selected in this review, which ranged from 73.3% to 95.2% (Xu *et al*., 2016; Feichtinger *et al*., 2017; Ho *et al*., 2018; Vera-Rodriguez *et al*., 2018; Yeung *et al*., 2019).

## CONCLUSION

The results found report successful amplification, high agreement rates and good sensitivity and specificity of the culture medium analysis. We can conclude that the time the embryo remains in contact with the culture medium, the need for complete removal of cumulus cells and the combination of blastocele fluid with spent culture medium favored the results.

However, NiPGT does not yet have enough reliability to replace PGT in assisted reproduction clinics, but it has many advantages when compared to the traditional invasive method, such as the easy methodology without the need for embryo manipulation and the screening of embryos that do not have viable morphology for biopsy. In order to elucidate all the issues raised in this study, it is necessary to agree on the chromosomal amplification and analysis methodology used, in order to define the underlying causes of the disagreement with the results of the embryo biopsy samples. In addition to standardizing protocols for media collection. More studies should be carried out using larger samples, so that we can better understand the mechanisms of DNA origin and release in the medium.
